# Molecular Designs for Enhancement of Polarity in Ferroelectric Soft Materials

**DOI:** 10.1038/srep16606

**Published:** 2015-11-16

**Authors:** Ryo Ohtani, Manabu Nakaya, Hitomi Ohmagari, Masaaki Nakamura, Kazuchika Ohta, Leonard F. Lindoy, Shinya Hayami

**Affiliations:** 1 Department of Chemistry, Graduate School of Science and Technology, Kumamoto University, 2-39-1 Kurokami, Chuo-ku, Kumamoto, 860-8555, Japan; 2 Department of Functional Polymer Science, Faculty of Textile Science and Technology, Shinshu University, Ueda 386-8567, Japan; 3 School of Chemistry, The University of Sydney, NSW 2006, Australia; 4 Institute of Pulsed Power Science (IPPS), Kumamoto University, 2-39-1 Kurokami, Chuo-ku, Kumamoto, 860-8555, Japan

## Abstract

The racemic oxovanadium(IV) salmmen complexes, [VO((rac)-(4-X-salmmen))] (X = C_12_C_10_C_5_ (**1**), C_16_ (**2**), and C_18_ (**3**); salmmen = N,N′-monomethylenebis-salicylideneimine) with “banana shaped” molecular structures were synthesized, and their ferroelectric properties were investigated. These complexes exhibit well-defined hysteresis loops in their viscous phases, moreover, **1** also displays liquid crystal behaviour. We observed a synergetic effect influenced by three structural aspects; the methyl substituents on the ethylene backbone, the banana shaped structure and the square pyramidal metal cores all play an important role in generating the observed ferroelectricity, pointing the way to a useful strategy for the creation of advanced ferroelectric soft materials.

Ferroelectric materials have attracted much attention for use in the development of data storage, sensors, mechanical actuation devices and other optoelectronic applications^1^,^2^,^3^,^4^,^5^,^6^,^7^. Ferroelectricity not only occurs in solid materials such as metal oxides^8^,^9^, polymers^10^ and organic molecules^11^,^12^ but can also be present in soft materials incorporating long alkyl chains^13^. The incorporation of polarity in molecular structures is an important design issue when generating new ferroelectric materials. For solid materials, off-centre displacements of constituent structural elements such as metal ions and protons results in polarity that gives rise to ferroelectrics^8^,^9^,^10^,^11^,^12^,^13^. On the other hand, fluidic soft materials usually preferentially form molecular alignments that cancel molecular dipole moments, as a consequence the design of fluid systems incorporating polarity remains a significant challenge. Up to now, an introduction of chiral alkyl chains and the design of banana shaped molecules have been applied to overcome this challenge, leading to weak ferroelectricity being observed in viscous liquid crystalline phases^14^,^15^,^16^,^17^,^18^,^19^,^20^,^21^,^22^,^23^.

The design and construction of banana shaped molecules provides a powerful approach for developing new ferroelectric soft materials including for achiral or racemic systems^16^,^17^,^18^,^19^,^20^,^21^,^22^,^23^. Several such bent organic compounds incorporating terminal bulky groups such as long alkyl chains have been synthesized that resulted in persisting macroscopic polarity accompanied by ferroelectric properties; however, their reported ferroelectric polarizations were very weak. In a bid to enhance the ferroelectricity of such fluidic soft materials, we^24^ and others^25^,^26^,^27^ have focused on a strategy in which a polarized metal complex incorporating a square pyramidal coordination geometry is incorporated into molecule cores. In particular, oxovanadium (VO) complexes have distorted square pyramidal geometries^25^,^28^,^29^ that would enhance the macroscopic polarity. Such a distorted square pyramidal arrangement is not found in organic compounds. Thus the approach is to combine a banana shaped molecular structure with a suitable square pyramidal metal core so that they act synergistically to induce enhanced ferroelectric properties.

In this study, we synthesized three banana shaped oxovanadium (VO) complexes, [VO(4-X-salmmen)], incorporating racemic N,N′-monomethylenebis-salicylideneimine (salmmen) ligands incorporating bulky alkoxy substituents (X) at the 4-positions of the aromatic rings (Fig. 1). These vanadium(IV) complexes each showed wide P – E hysteresis loops for their viscous phases. We have probed the roles of their respective structural features on the corresponding ferroelectric properties by comparing the results with those for analogs that have rod shape and a square planar coordination geometries.

## Results

### 

**Figure 2:**
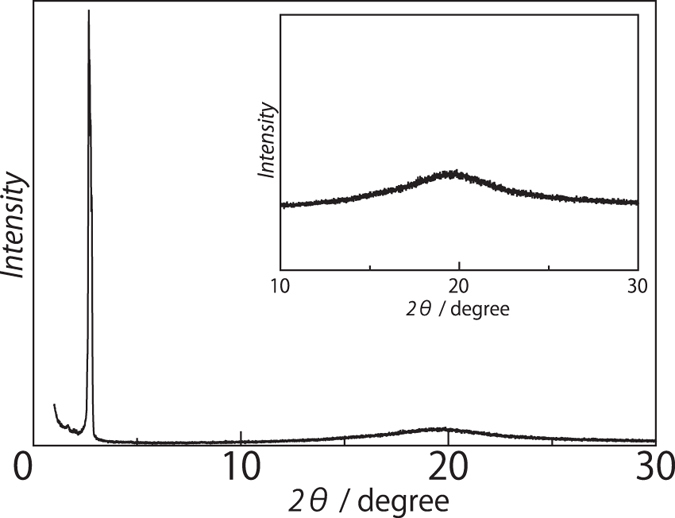
PXRD pattern for the LC phase of 1 at 298 K.

The new banana shaped racemic VO compounds [VO(4-X-salmmen)] (X = C_12_C_10_C_5_ (**1**), C_16_ (**2**) and C_18_ (**3**)) incorporating branched and single alkyl chains were synthesized by complexation between VOSO_4_•nH_2_O and (*rac*)-4-X-salmmen^30^,^31^,^32^. Final products were obtained after purification by column chromatography (SiO_2_, CHCl_3_). Compound **1** is viscous oil while **2** and **3** are solids at room temperature. Powder X-ray diffraction spectroscopy (PXRD) results demonstrated that **1** is in a liquid crystal (LC) state at 298 K for which sharp peaks in the low angle region and broad scattering halos in the wide angle region were observed; this corroborated its LC nature for which the *d* spacing value was 33.0 Å (2*θ* = 2.68°) (Fig. 2). In contrast, **2** and **3** are crystalline phases at 298 K. The branched alkyl chains in **1** likely play a role in decreasing the phase transition temperature of **1**.

The phase transition behaviour of **1**–**3** has been investigated by differential scanning spectroscopy (DSC). **1** shows a phase transition from a crystalline state to the LC state at 208.3 K on heating, with no transition from the LC to an isotropic liquid (IL) state being observed in the DSC curve (Fig. S1). Compounds **2** and **3** also exhibited multiphase natures, with phase transitions accompanied by translocation of the alkyl chains at 314.6 K and 379.1 K for **2** and at 329.6 K, 358.7 K and 367.4 K for **3**, with melting to IL states at 412.2 K for **2** and 413.5 K for **3**, respectively. However, their LC properties were not able to be obtained, as shown by the results from variable temperature PXRD in which we observed remaining sharp peaks in the middle and wide angle regions at 410 K after annealing treatments at 443 K and sequential slow cooling in each case (Fig. S2).

We measured the dielectric constants of **1**–**3** within the frequency range 100 Hz to 1 kHz by inductance capacitance and resistance (LCR) measurements in order to investigate the electric field responses to the phase transitions (Fig. S3). During the heating of **1** (starting from 300 K), the dielectric constant remained unchanged up to 350 K. Subsequently, an abrupt elevation of the dielectric constant occurred above 350 K in **1** in accord with a transformation (melting) occurring from the LC of **1** then to its IL state (even though an endothermic peak was not observed in the DSC curve). In the case of **2** and **3**, anomalies were observed at 356 K, 390 K and 418 K for **2**, while, for **3**, anomalies were observed at 356 K, 366 K, 370 K, and 404 K, respectively, with these transitions being consistent with the results of the DSC analysis mentioned above. A dielectric response at 1 kHz showed a similar trend with lower peak values, thus demonstrating that the dipoles of the complexes are insensitive to an electric field oscillating at the above frequency.

**Figure 3:**
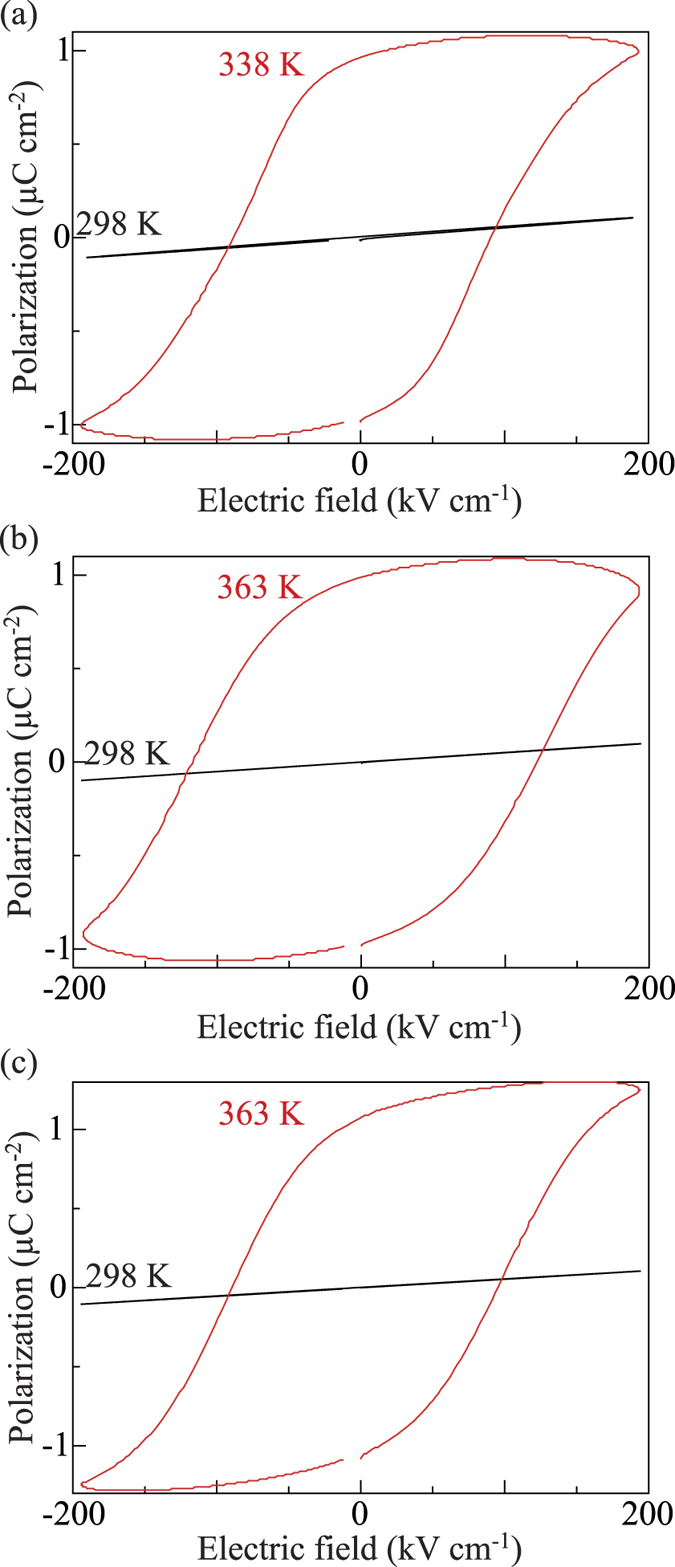
Hysteresis loops arising from the electric field dependent dielectric polarization of (a) 1, (b) 2 and (c) 3.

The ferroelectric properties of each of the phases observed for **1**, **2**, and **3** were studied by analysing polarization vs. electric field (P – E) curves using a TF Analyzer1000. All compounds failed to show ferroelectric behaviour at 298 K. Ferroelectricity of **1** was developed at 338 K, while **2** and **3** at 363 K showed well-defined hysteresis behaviour (Fig. 3). In the case of **1**, the spontaneous polarization (Ps) is 1.08 μC cm^−2^ under a coercive 100 kV cm^−1^ field with *E*_*c*_ at 50 Hz. A strong frequency relaxation occurred at −194 kV cm^−1^, which is characteristic of ferroelectric behaviour. Compounds **2** and **3** exhibited almost identical (ferroelectric) behaviour, with a Ps of 1.03 μC cm^−2^ and 1.09 μC cm^−2^, respectively, under a coercive 100 kV cm^−1^ field with *E*_*c*_ at 150 Hz and a relaxation at −194 kV cm^−1^. Hysteresis loops were not observed above the melting points.

## Discussion

**Figure 4:**
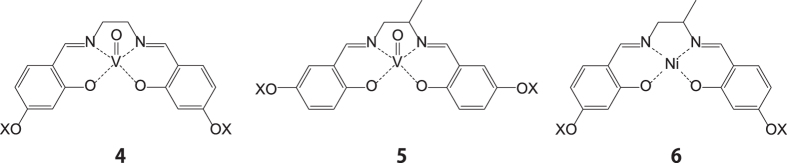
Molecular structures of 4–6.

We have investigated the origins of the above ferroelectricity in terms of structural aspects of (a) the methyl substituent on the ethylene backbone, (b) the banana shaped structure and (c) the square pyramidal coordination geometry of VO by synthesizing five ‘control’ compounds of the R and S derivatives **1X** (X = R or S) (Fig. S4), a [VO(4−C_16_−salen)] (**4**) incorporating a salen ligand instead of the salmmen ligand, a rod shaped racemic [VO(5−C_16_−salmmen)] complex (**5**)^24^ and a racemic [Ni(4−C_16_−salmmen)] complex (**6**) incorporating a square planar nickel ion instead of the vanadyl (V = O) group (Fig. 4 and Table 1). Both **1R** and **1S** showed very similar ferroelectric properties to **1** (Fig. S5); however, **4** without a methyl substituent as a chiral centre on the ethylene backbone showed only weak spontaneous polarization (67 nC cm^−2^) despite its banana shaped molecular structure. On the other hand, the rod shaped **5** bearing a methyl substituent also showed weak ferroelectricity, demonstrating that a coexistence of the factors (a) and (b) are important for observing the ferroelectricity. Moreover, we found that the presence of (c) the square pyramidal coordination geometry of the VO centre also plays important role in the generation of ferroelectricity. The ferroelectricity of the square planar derivative **6** incorporating both a methyl substituent and a banana shaped structure was also weak (53 nC cm^−2^). From these results, we conclude that synergism arising from the three principal structural features given by (a), (b) and (c) is important for the development of the ferroelectricity observed in **1–3** (Table 1).

Ferroelectricity for **1** developed at 338 K, a lower temperature than for **2** and **3**, reflecting its phase transition occurring at lower temperature and in accord with the presence of branched alkyl chains. Compound **1** is in the LC state at both 298 K and 338 K, however, hysteresis was not observed at 298 K. This result indicates that a dipole inversion of the banana shaped **1** requires higher thermal energy than available at 298 K on application of the electric field. Although **2** and **3** do not show liquid crystal properties, their temperature-dependent softness and viscosity nature contribute to the generation of their ferroelectricity. These findings clearly open doors for the design and synthesis of the next generation of multifunctional ferroelectric soft materials.

## Conclusions

We have synthesised the banana shaped ferroelectric VO soft materials **1–3** employing racemic salmmen ligands bearing branched/linear long alkyl chains. These compounds exhibit ferroelectric properties associated with large spontaneous polarisations. Moreover, the substitution of branched alkyl chains in the banana shaped structure led to both development of liquid crystallinity at room temperature as well as a decrease of the ferroelectric phase transition temperature. The synthesis of metallomesogens exhibiting liquid crystallinity at room temperature, such as **1**, are anticipated to contribute not only to the development of optoelectronic display devices in applied electronics but also to the generation of soft materials exhibiting ferroelectricity at room temperature. Compounds **2** and **3** also show significant promise for exhibiting liquid crystallinity, and their possible simultaneous liquid crystallinity and ferroelectricity are under investigation. Clearly the construction of banana shaped square pyramidal metal complexes that also incorporate long alkyl chains provides a powerful strategy for generating novel multifunctional molecular soft materials displaying enhanced ferroelectricity.

## Methods

### Synthesis

#### 4-Hexadecoxysalicylaldehyde (4-C_16_-salicylaldehyde)

2,4-Dihydroxybenzaldehyde (2.0 g, 15 mmol), KHCO_3_ (1.8 g, 18 mmol), KI (0.50 g, 3.0 mmol) and 1-bromohexadecane (4.8 g, 16 mmol) were refluxed in absolute acetone for 2 days under Ar. Subsequently, after a filtration while hot, the filtrate was neutralized with aqueous HCl and extracted with chloroform (100 mL). The purple solid was purified on a silica gel column with hexane and chloroform (1:3, v/v). A white solid product was obtained; yield 3.9 g (75%). ^1^H NMR: (500 MHz, CDCl_3_:δ = 11.46 (s, 1H, COH), 9.67(s, 1H, OH), 7.40 (m, 1H, CH = CH), 6.51 (m, 1H, CH = CH), 6.39 (s, 1H, CH = C−), 3.98 (t, 2H, O−CH_2_), 1.77(sex, 2H, −CH_2_−), 1.23(m, 26H, −CH_2_−), 0.86 (t, 3H, CH_3_−)).

#### 4-Octadecoxysalicylaldehyde (4-C_18_-salicylaldehyde)

This compound was synthesized by the same method employed for 4-C_16_-salicylaldehyde using 1-bromooctadecane; yield 4.5 g (80%), ^1^H NMR: (500 MHz, CDCl_3_:δ = 11.42 (s, 1H, COH), 9.66(s, 1H, OH), 7.41 (m, 1H, CH = CH), 6.55 (m, 1H, CH = CH), 6.38 (s, 1H, CH = C−), 3.97 (t, 2H, O−CH_2_), 1.77(sex, 2H, −CH_2_−), 1.23(m, 30H, −CH_2_−), 0.87 (t, 3H, CH_3_−)).

#### 4-C_5_C_12_C_10_-salicylaldehyde

This compound was synthesized by the same method employed for 4-C_16_-salicylaldehyde using 5-decyl-1-bromoheptadecane; yield 1.6 g (21%), ^1^H NMR: (500 MHz, CDCl_3_:δ = 11.47 (s, 1H, COH), 9.68 (s, 1H, OH), 7.42 (m, 1H, CH = CH), 6.53 (m, 1H, CH = CH), 6.39 (s, 1H, CH = C−), 3.98 (t, 2H, O−CH_2_), 1.77(sex, 4H, −CH_2_−), 1.23(m, 43H, −CH_2_−), 0.86 (t, 6H, CH_3_−))

#### (*rac*)-4-C_16_-salmmen

4-C_16_-salicylaldehyde (0.83 g, 2.3 mmol) and (rac)-1,2-diaminopropane (0.15 g, 1.6 mmol) in MeOH (200 mL) were heated at 60 °C for 2 hours. The solution was distilled under reduced pressure and purified by chromatography on a silica gel column with hexane and ethyl acetate (3:1, v/v) as eluent. A yellow solid product was obtained; yield 0.55 g (62%), ^1^H NMR: (500 MHz, CDCl_3_:δ = 8.21 (s, 1H, CH = N−), 8.17 (s, 1H, CH = N−), 6.83–6.77 (m, 4H, CH = CH), 6,68 (s, 2H, CH = C−), 3.79 (t, 4H, O−CH_2_), 3.66–3.59 (m, 2H, CH_2_−C−), 1.65 (sex, 4H, −CH_2_−), 1.33–1.31 (m, 1H, CH−N), 1.24–1.16 (m, 55H, −CH_2_−, CH_3_−), 0.81 (t, 6H, CH_3_−)).

#### (*rac*)-4-C_18_-salmmen

This compound was synthesized by the same method as described for (*rac*)-4-C_16_-salmmen using 4-C_18_-salicylaldehyde; yield 0.56 g (68%), ^1^H NMR: (500 MHz, CDCl_3_:δ = 8.22 (s, 1H, CH = N−), 8.15 (s, 1H, CH = N−), 6.83–6.76 (m, 4H, CH = CH), 6,68 (s, 2H, CH = C−), 3.79 (t, 4H, O−CH_2_), 3.65–3.59 (m, 2H, CH_2_−C−), 1.65 (sex, 4H, −CH_2_−), 1.33–1.31 (m, 1H, CH−N), 1.26–1.16 (m, 61H, −CH_2_−, CH_3_−), 0.82 (t, 6H, CH_3_−)).

#### (*rac*)-4-C_5_C_12_C_10_-salmmen

This compound was synthesized by the same method as described for (*rac*)-4-C_16_-salmmen using 4-C_5_C_12_C_10_-salicylaldehyde; yield 0.32 g (31%), ^1^H NMR: (500 MHz, CDCl_3_:δ = 8.22 (s, 1H, CH = N−), 8.15 (s, 1H, CH = N−), 6.83–6.76 (m, 4H, CH = CH), 6,68 (s, 2H, CH = C−), 3.79 (t, 4H, O−CH_2_), 3.65–3.59 (m, 2H, CH_2_−C−), 1.65 (sex, 8H, −CH_2_−), 1.33–1.31 (m, 1H, CH−N), 1.26–1.16 (m, 89H, −CH_2_−, CH_3_−), 0.82 (t, 12H, CH_3_−))

#### [VO(*rac*)-4-C_5_C_12_C_10_-salmmen] (1)

(*rac*)-4-C_5_C_12_C_10_-salmmen (0.51 g, 0.48 mmol), VOSO_4_•nH_2_O (0.15 g, 0.92 mmol) and pyridine (1.5 mL) in MeOH (250 mL) was heated at 60 °C for 3 hours. The solution was distilled under reduced pressure and purified by column chromatography (SiO_2_, CHCl_3_). Green oil, yield 0.37 g (67%), Anal. Calcd. for C_71_H_124_N_2_O_5_V: C, 75.02; H, 11.00; N,2.46. Found: C, 75.14; H, 11.24; N, 2.58.

#### [VO((*rac*)-4-C_16_-salmmen)] (2)

This complex was synthesized by the same method as employed for **1** using (*rac*)-4-C_16_-salmmen. Green powder, yield 0.26 g (65%), Anal. Calcd. for C_49_H_80_N_2_O_5_V: C, 71.07; H, 9.74; N,3.38. Found: C, 70.81; H, 9.99; N, 3.54.

#### [VO(*rac*)-4-C_18_-salmmen] (3)

This complex was synthesized by the same method as employed for **1** using (*rac*)-4-C_18_-salmmen. Green powder, yield 0.34 g (81%), Anal. Calcd. for C_53_H_88_N_2_O_5_V : C, 71.99; H, 10.03; N,3.17. Found: C, 71.69; H, 10.31; N, 3.37.

### Physical measurements

^1^H NMR spectra were recorded on a JEOL (500-ECX) instrument (500 MHz) in deuterated solvents using TMS as internal reference. Elemental analyses (C,H,N) were carried out on a J-SCIENCE LAB JM10 at the Instrumental Analysis Centre of Kumamoto University. Differential scanning calorimetry (DSC) thermal analysis was carried out at 5 K min^−1^ on a SHIMADZU DSC50. Powder X-ray diffraction (PXRD) measurements were performed on a Rigaku X-ray diffract meter RAD-2A with a 2.0 kW Cu Ka X-ray. Dielectric constants were measured by an inductance capacitance and resistance (LCR) meter on a Wayne Kerr 6440B LCR meter. The determination of polarization was performed on an aixACT TF analyser 1000. Circular dichroism (CD) spectra of KBr pellets containing samples were measured by JASCO J-820 at RT.

## Additional Information

**How to cite this article**: Ohtani, R. *et al.* Molecular Designs for Enhancement of Polarity in Ferroelectric Soft Materials. *Sci. Rep.* **5**, 16606; doi: 10.1038/srep16606 (2015).

## Supplementary Material

Supplementary Information

## Figures and Tables

**Figure 1:**
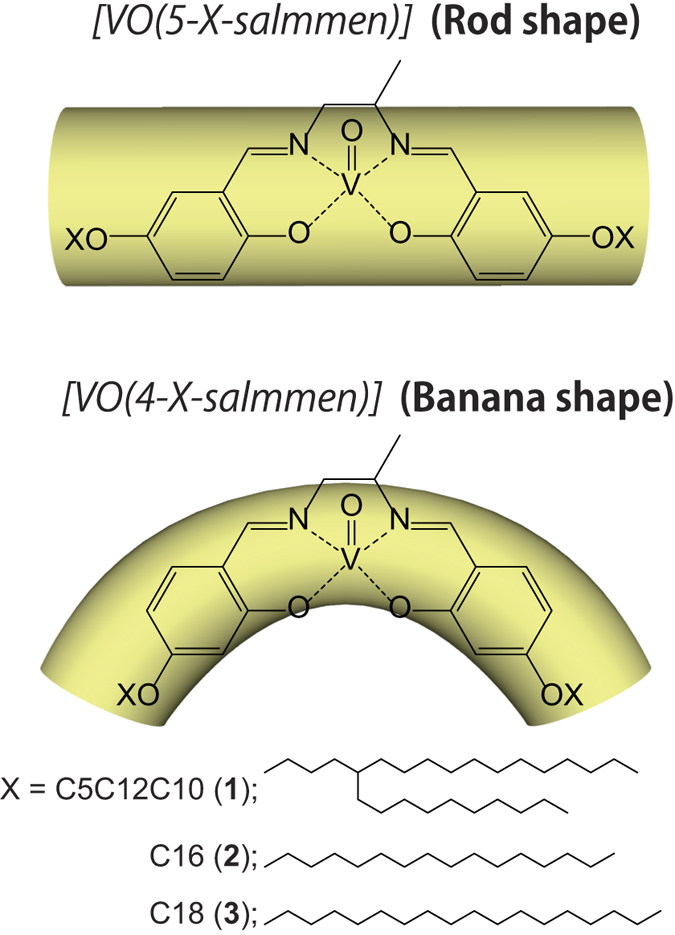
Molecular design of the banana shaped oxovanadium(IV) complexes.

**Table 1:** Relationship between the three structural aspects in **1–6** and the observed ferroelectricity.

	Methyl substituent	Banana shape	Pyramid shape	Ferroelectricity
**1–3**	✓	✓	✓	Strong
**4**		✓	✓	Weak
**5**	✓		✓	Weak
**6**	✓	✓		Weak
