# Supplementary material for: Molecular Designs for Enhancement of Polarity in Ferroelectric Soft Materials
Source: Sci Rep. 2015 Nov 16;5:16606. doi: 10.1038/srep16606 (PMC4645115; doi:10.1038/srep16606)
Supplement: Supplementary Information [file srep16606-s1.pdf]

Supporting Information

Molecular Designs for Enhancement of Polarity in Ferroelectric Soft Materials

Ryo Ohtani, Manabu Nakaya, Hitomi Ohmagari, Masaaki Nakamura, Kazuchika Ohta, Leonard F. Lindoy, and Shinya Hayami

Corresponding authors:
S. Hayami, hayami@sci.kumamoto-u.ac.jp

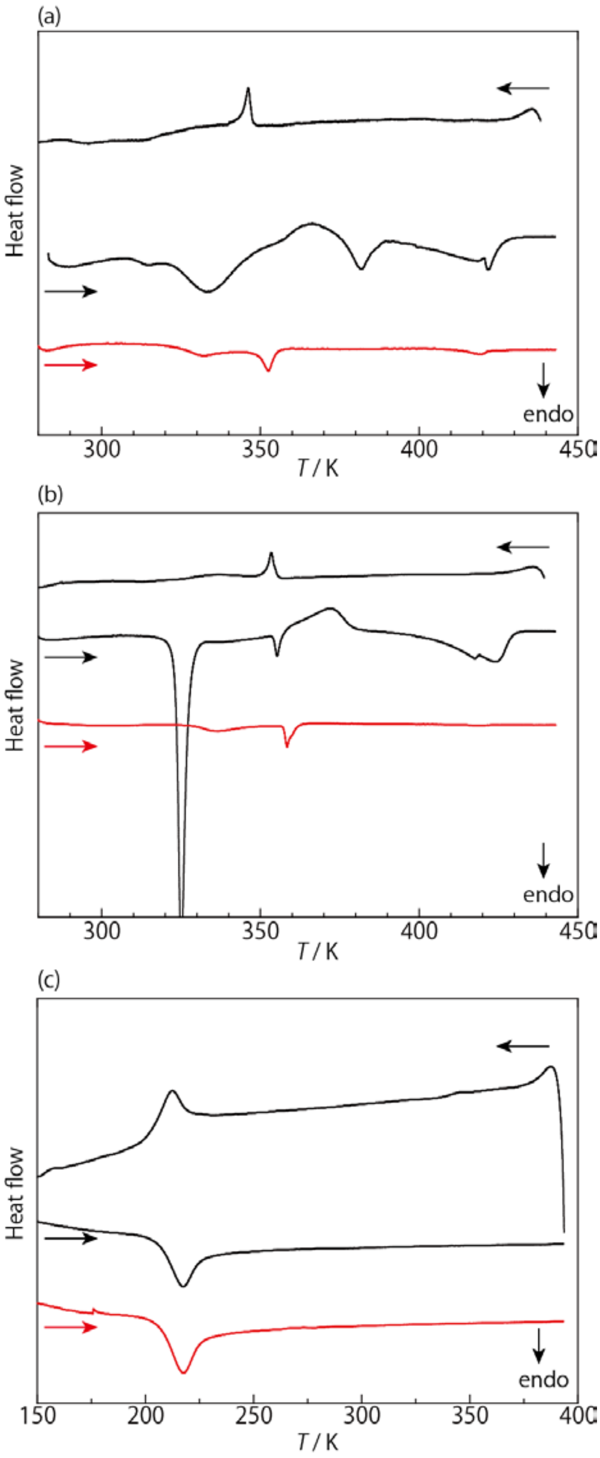

Figure S1 DSC curves for (a) **1**, (b) **2** and (c) **3**. (black line: the first cycle, red line: the second cycle)

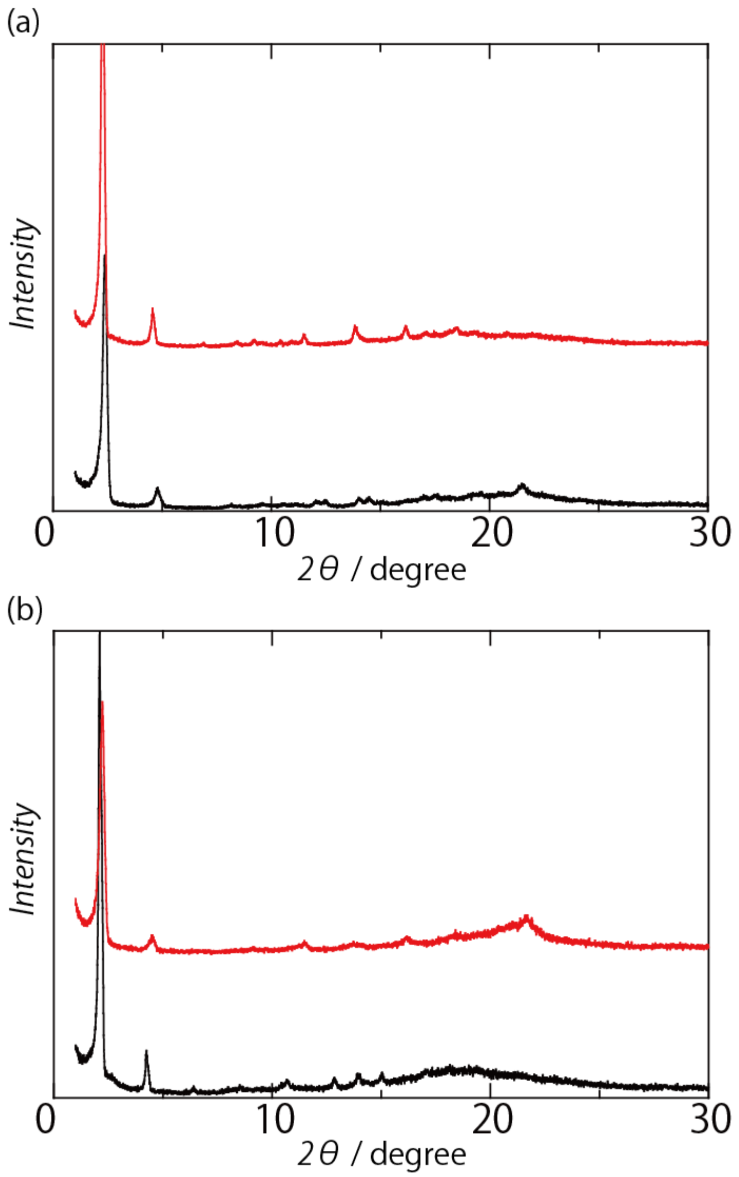

Figure S2 Valuable temperature of PXRD of (a) **2** and (b) **3**. (red line: 410 K, black line: 298 K)

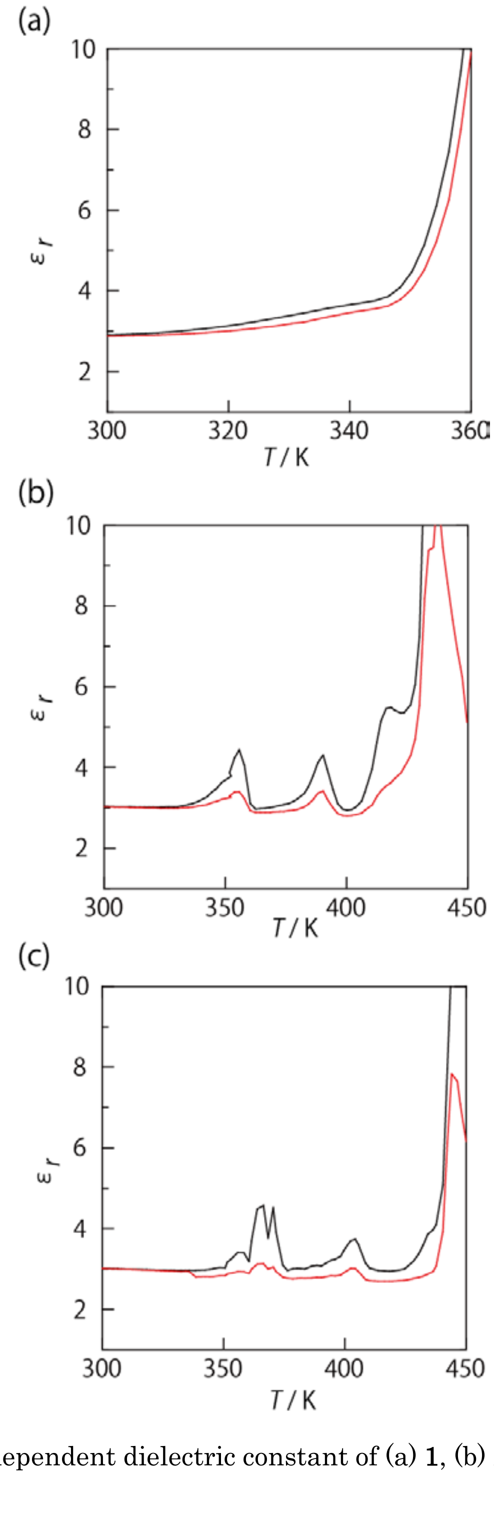

Figure S3 Temperature dependent dielectric constant of (a) **1**, (b) **2** and (c) **3**. (black line: 100 Hz, red line: 1 kHz)

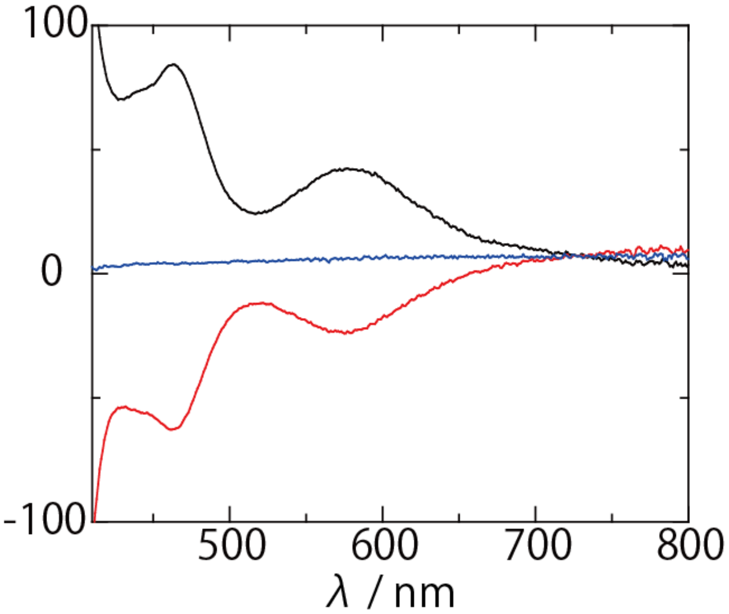

Figure S4 CD spectra for **1**. (black line: R, red line: S, blue line: racemic)

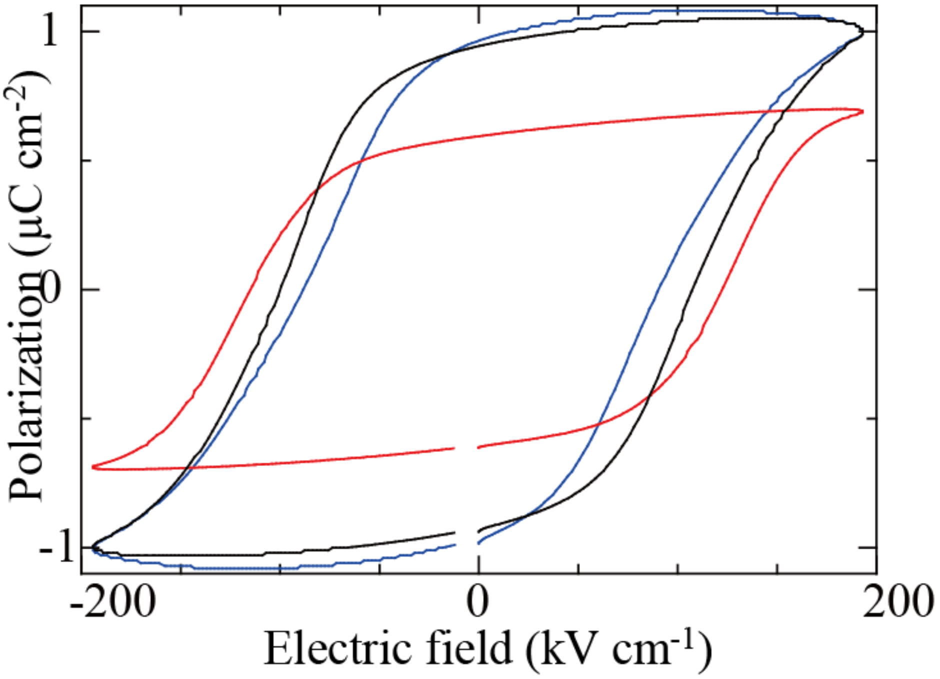

Figure S5 $P-E$ hysteresis loops for **1X**. (black line: R, red line: S, blue line: racemic)
